# A model to understand risk behavior: Interoception awareness and motivational systems in the binge watching.

**DOI:** 10.1016/j.heliyon.2025.e42253

**Published:** 2025-01-27

**Authors:** Giuseppe Forte, Francesca Favieri, Francesca Agostini, Maria Casagrande, Renata Tambelli

**Affiliations:** a Department of Dynamic, Clinical Psychology and Health Studies, “Sapienza” University of Rome, Via degli Apuli 1, 00185, Rome, Italy; b Department of Psychology, “Sapienza” University of Rome, Via dei Marsi 78, 00185, Rome, Italy

**Keywords:** Interoception, Binge watching, Body awareness, Emotion regulation, Behavior addiction

## Abstract

This study aims to test the interoceptive-motivational hypothesis as a possible marker for the risk of developing binge-watching (BW) as a behavioral addiction. Like other risky behaviors, BW can be included in a model that includes the interaction of personological and physiological factors as predictors of the behavioral outcome. On a sample of 741 young adults, a structural equation model considered the association between the interoceptive indices (MAIA questionnaire), inhibition/activation systems (BIS/BAS questionnaire) of the motivational theory, and BW pattern (BWAQ questionnaire). The results suggested a different interaction between the variables when BW was considered as a leisure activity and as an at-risk behavior. While in the first case, interoception and BIS/BAS systems interact, and BIS and interoception positively affect the increase of BW as a leisure activity, in BW as at-risk behavior, the interoceptive-motivational link is lost, and a different pattern of association with the behavior emerges. BIS and BW are still positively associated with the problematic expression of the behavior. The result would suggest that persons exhibiting better interoceptive sensitivity display lower risk of addictive BW. Finally, the interoceptive-motivational model, if confirmed in other at-risk behaviors, would provide a new perspective in the field of behavioral addictions.

## 1 Introduction

In recent decades, neuroscience has focused attention on clarifying the inner world and how it interacts with the external world through structural integrations, functional systems, and behavioral pathways [1]. For a long time, researchers have focused on the sensory side – i.e., the information coming from the external world – suggesting an exteroceptive perspective from the outside to the inside. However, the amount of information coming from within the body has generally been neglected, relegating the role of the interoceptive system as secondary in the behavioral response [2].

Interoception involves the body-to-brain axis of sensation concerning the state of the internal body state [[3], [4], [5]]. The interoceptive process originates from the integration of different types of sensory information that leads to the awareness of bodily sensations (e.g., pain, touch, temperature) and allows the organism to regulate its internal state homeostatically [6]. Starting with James, the role of bodily feedback has been extended to the cognitive and behavioral domain [7], suggesting that behavioral responses are aimed at maintaining homeostasis through the translation of internal information. In this view, interoception helps to maintain homeostatic balance involving multiple sources at neural, behavioral, and higher-order cognitive functions (e.g., decision-making) that continually predict the state of the body and act by reducing self-control failures. Thus, in the presence of low interoceptive ability (i.e., interoceptive sensitivity), characterized by an inability to detect, discriminate, and appropriately name body sensations, engagement in impulsive behaviors may occur as a maladaptive consequence of arousal handling [8,9], predisposing to impulsive behaviors [10]. In turn, impulsivity is associated with risky behaviors due to the high sensitivity to reward and difficulty with inhibition. In this sense, two brain systems are sensitive to punishment and reward and help in controlling individual behavior, including impulsivity traits: the Behavioral Inhibition System (BIS) and the Behavioral Activation System (BAS) [11]. While the BIS is the expression of the system of motivational regulation, sensitive to punishment and reduction or termination of reward, inhibiting behavior associated with negative consequences, the BAS regulates appetitive motivation and is activated by rewards and by the reduction or termination of punishment, increasing approach behavior and positive emotion. Accordingly, the balance between BIS and BAS seems to be fundamental for behavioral control, and a growing body of evidence confirms the association between the unbalance in its subdomains and risky behavior [12], which can lead to a persistent approach despite of the awareness of negative consequences and can lead to behavioral addiction (BA) [13]. Although BA involves an alteration of the dopamine system, the motivation, and emotional salience (both positive and negative) of the behavior play a significant role in the implementation of addiction. In this sense, it is plausible that interoceptive processes, as well as BIS/BAS responses, may be involved in the onset and maintenance of both substance and behavioral addiction [14]. However, especially in risk behaviors that can exacerbate BA, the questions “How do interoceptive sensitivity and motivational systems relate to each other and to risk behavior?” and “Could an interoceptive-motivational system predict the risk of developing BA?” remain unanswered.

Accordingly, we chose to test an interoceptive-motivational model in the frame of a common and novel risk behavior in the young population, i.e., binge-watching (multiple viewing of TV-series content), which has been reported to have BA characteristics in some circumstances, but that is commonly included in a functional continuum from adaptive (leisure activity) to maladaptive (BA) [15,16].

In fact, previous studies [[15], [16], [17], [18], [19], [20]] show that BW can be viewed as a practice that impacts mental health through a continuum from helpful to harmful. The beneficial effects of BW are due to its entertainment function, which helps viewers to reduce negative feelings (e.g., stress, anxiety, and mood alteration), while the addictive-like features of BW mentioned above lead to a worsening of mental health and an increase in perceived distress. However, the reasons why behavioral trajectories may be included in this continuum remain unknown. Therefore, examining the relationship between interoception and motivation within the activation/inhibition system framework, considering both the potentially addictive and pleasurable nature of this behavior, may clarify the background of the risk behavior.

Given the previously reported association between interoceptive sensitivity indices and BIS/BAS domains [12], their influence on behavioral outcomes, and the complex relationship with risk behavior [10], we expect that these two dimensions may cause different expressions of BW. Specifically, we predict distinct relationships that may represent markers of behavioral outcomes on the positive and potentially negative sides of the continuum (i.e., from leisure to pathological binge-watcher).

**Fig. 1:**
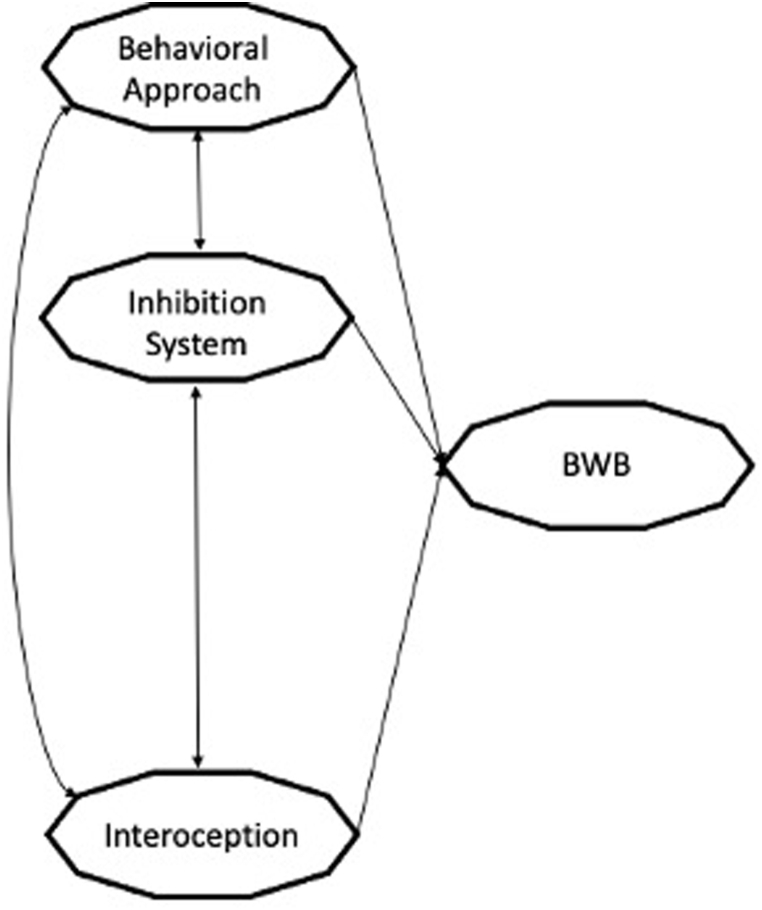
The interoceptive-motivational model of BW.

Considering previous studies, we speculated that the interplay between interoceptive sensitivity and motivational systems of BIS/BAS might explain TV series viewing behavior in its continuum. Differences between people who watch TV series as a pleasure activity or in a compulsive manner are conceptualized in this sense as qualitative rather than quantitative (i.e., absence/presence of relationship). Accordingly, we hypothesized that higher interoceptive ability might be associated with higher BW as a leisure activity and lower BW as a risk behavior. Instead, the motivational systems will always be related to behavioral expression because of the role of motivation in reducing the negative effects of punishment through approach behavior. Furthermore, different relationships between interoceptive ability and motivational systems are expected. This model diverges from previous ones since it focuses on the fundamental incentives that potentially drive individuals to behaviors. Interoceptive-motivational model offers a novel perspective compared to existing models of behavioral addiction, and generally in behavioral models furnishing insights on their expressions, since integrating suggesting as their expression may represent a way of regulating internal or physical sensations (e.g., stress or anxiety) which are perceived through interoception. Cognitive dimensions both cold and hot beyond the motivational system may interact with the elaboration of body experience and its interpretation, justifying possible dysregulation and the differences between leisure and problematic activities. Current research revalues mind-body interaction and in this perspective, this study aims to furnish further support to it for both researchers and clinicians. In this sense it could permit a better understanding of the individuals behind the behavior, moving away from confirmatory approach and towards a process based approach. In this sense, a confirmation of the model would provide us with a support in the direction of reducing the overpathologization of all the behaviors, but defining what and in how implicit and explicit individuals’ characteristics may affect their negative decline [15]. The conceptual model is summarized in Fig. 1.

## 2 Method

### 2.1 Participants

Seven hundred forty-one young adults who completed an online survey, addressed to TV series viewers, were included in the study (mean age = 24.25; SD = 3.36; 65.18 % females). Exclusion criteria of the recruitment included psychopathological, psychiatric, or chronic medical conditions that may affect interoceptive awareness of BIS/BAS balance (n = 25 excluded).

### 2.2 Instruments

The *Binge-Watching Addiction Questionnaire* (BWAQ; [16]) was administered to assess the watching style behavior. The BWAQ is a self-report questionnaire consisting of 24 items on a 5-point Likert scale (i.e., from 0 = never to 4 = always) that has good reliability in the Italian population (Cronbach's α = 0.94). The BWAQ provides a global score of BW behavior. Based on this score, it is possible to determine the degree of dysfunctional behavior: scores above 50 are in the normal range, while a score equal to or greater than 51 indicates mild to relevant problematic behavior. In addition to a global score, the BWAQ also provides an assessment of different components of the addiction-like characteristics of BW: (i) Craving: expresses the degree of pleasure experienced during the behavior, is associated with worry and discomfort about bodily sensations; (ii) Dependency: refers to the compulsive component of the BW; (iii) Anticipation: indicates the constant tendency to search for TV content; and (iv) Avoidance: focuses on the dimension of minimizing BW as a problem.

The Italian version of the *Multidimensional Assessment of Interoceptive Awareness* (MAIA [21,22]; was administered to assess interoceptive awareness and sensitivity. The MAIA is a 32-item self-report questionnaire on a 6-point Likert scale (i.e., from 0 = never to 5 = always) that allows the assessment of eight domains of interoceptive awareness. The domains evaluated are (1) noticing (i.e., awareness of own's bodily sensations); (2) not-distracting (i.e., the tendency not to ignore negative sensations); (3) not-worrying (i.e., the tendency to not experience emotional distress when negative sensations are experienced); (4) attention regulation (i.e., the ability to control and orient attention to own's bodily sensations); (5) emotional awareness (i.e., the awareness of the body sensation and emotional states connection); (6) self-regulation (i.e., the ability to regulate psychological distress through attention to bodily sensations); (7) body listening (i.e., the tendency to listen to one's body for insight actively); (8) trusting (i.e., the experience of one's body as trustworthy). Each subscale scored satisfactorily (Cronbach's α ranging from 0.53 to 0.80).

Higher scores in each subscale indicate higher levels of awareness according to the different domains.

The *Behavior Motivation System Scale* (BIS-BAS; [11 is a self-reported questionnaire composed of 24 items on a 4-point Likert scale (1 = very true for me; 4 = very false for me). The scale includes one subscale to assess BIS (behavioral inhibition system) and three subscales to assess the three components of BAS: drive (the motivation to follow one's goals), reward responsiveness (the sensitivity to pleasant reinforcers in the environment), and fun-seeking (the motivation to find novel rewards spontaneously). The reliability of the instrument was adequate for both domains (Cronbach's α for the BIS and BAS, respectively, of 0.72 and 0.74).

### 2.3 Procedure

A survey including measures of binge-watching, interoceptive sensitivity and motivational systems was disseminated online to recruit young people 18 years of age and older that are involved in experience of watching TV series. The availability of the survey was consequent upon the acceptance of the informed consent form that outlined the main aims of the study. The average duration of the survey was 20 min. Data were collected anonymously from April to June 2022, and no sensitive data were collected.

### 2.4 Ethics

The project was approved by the Ethics Committee of the Department of Dynamic, Clinical Psychology and Health, "Sapienza" University of Rome (protocol number 0000801). All methods were carried out in accordance with relevant guidelines and regulations and in line with the Declaration of Helsinki.

### 5.5 Data analysis

Participants were classified on the basis of their global score on the BW measure (e.g., Non-viewers, Leisure BW, and Problematic BW).

Initially, the Pearson correlation was calculated to analyze the association between the global score of the BWAQ and the scores on the MAIA subscales. In addition, Analyses of Variance were performed with the Group as the independent variable and each MAIA and BIS/BAS domain as dependent variables. This first data analysis was run with Jamovi 2.0. Significance was set at a p-value <0.05.

To test the substantive hypotheses of the study outlined in Fig. 1, we carried out a Structural Equation Modeling analysis (SEM) using the “lavaan” packages [23] for R. Interoceptive ability, motivation, and reward were measured using composite scores of each subscale and were considered as latent variables in the model.

The model was estimated using Robust Maximum Likelihood. We first performed a single group analysis to assess the reliability of the latent variables. Then, we tested the invariance of the model parameters between problematic BW and non-problematic BW. We assessed model fit using the Comparative Fit Index (CFI), Tucker-Lewis Index (TLI), Root Mean Square Error of Approximation (RMSEA), and Standardized Root Mean Square Residual (SRMR). A CFI and TLI value above 0.90 is considered acceptable [24]. A good fit is supported by RMSEA and SRMR lower than 0.06 and 0.08, respectively.

## 3 Results

### 3.1 Correlational analyses

Pearson's linear correlation coefficient was calculated for the association between MAIA subscales and BWAQ global score. Global BWAQ reported a significant positive correlation with the Noticing (r = 0.14; p = 0.001), Emotional Awareness (r = 0.13; p = 0.001), and Body Listening (r = 0.08; p = 0.001) subscales of MAIA. Moreover, a negative linear correlation between the global BWAQ and the Not Worrying subscale was reported (r = −0.15; p = 0.001).

Specific correlations are reported in Table 1.

**Table 1:** Pearson's linear correlation.

	BW-Total Score	BW-Craving	BW-Dependency	BW-Anticipation	BW-Avoidance
MAIA_Noticing	0.15∗∗∗	0.15∗∗∗	0.13∗∗∗	0.15∗∗∗	0.02
MAIA_Not-Distracting	−0.03	−0.01	−0.03	−0.10∗∗	−0.09^a^
MAIA-Not-Worring	−0.15∗∗∗	−0.14	−0.09	−0.13∗∗∗	−0.06
MAIA-Attention-Regulation	−0.03	−0.01	−0.06	0.04	0.05
MAIA-Emotional-Awarenss	0.13∗∗∗	0.13∗∗∗	0.08^a^	0.11∗∗	0.11∗∗
MAIA-Self regulation	0.04	0.05	−0.05	0.09^a^	0.08^a^
MAIA-Body Listening	0.08	0.10^a^	0.03	0.03	0.03
MAIA-Tusting	−0.03	−0.03	−0.07	0.07	−0.02
BAS_Drive	0.04	0.02	0.05	0.08^a^	0.01
BAS_FunSeeking	0.07	0.06	0.09^a^	0.05	−0.08^a^
BAS_Reward	0.05	0.04	0.09^a^	0.07	0.04
BIS	0.13∗∗∗	0.13∗∗∗	0.16∗∗∗	0.08^a^	0.03

a p < 0.05, ∗∗p < 0.01, ∗∗∗p < 0.001.

### 3.2 Analyses of the variance

ANOVA conducted between the groups (BW as a leisure activity and BW as at-risk behavior) did not report significant differences either in the MAIA or the BIS/BAS scales, only in the Body Listening subscale of the MAIA who adopted BW as a leisure activity showed higher scores than those who experienced BW as an at-risk behavior (see Table 2).

**Table 2:** Groups characteristics and ANOVA results.

	**Leisure BW**	**Problematic BW**	**F**	**p**
N (M/F)	726 (257/469)	16 (2/14)		
Age	24.2 (3.4)	23.8 (3.2)		
Global Score BWAQ (mean ± sd)	21.2 (11.2)	57.5 (6.1)		
**MAIA indices**				
Noticing	2.84 (1.26)	2.96 (1.21)	<1	0.69
Not Distracting	2.51 (1.03)	2.58 (0.97)	<1	0.78
Not worrying	2.16 (1.09)	2.40 (1.20)	<1	0.43
Attention Regulation	2.51 (1.14)	2.23 (0.86)	<1	0.32
Emotional Awareness	2.92 (1.28)	2.94 (1.19)	<1	0.97
Self Regulation	2.21 (1.26)	2.39 (1.06)	<1	0.57
Body Listening	2.96 (1.10)	2.19 (1.34)	5.12	0.02
Trusting	2.72 (1.45)	2.48 (1.23)	<1	0.51
BIS/BAS				
BAS Drive	11.64 (2.36)	11.68 (2.41)	<1	0.95
BAS Fun Seeking	11.62 (2.25)	11.18 (1.90)	<1	0.44
BAS Reward	16.81 (2.84)	16.36 (3.81)	<1	0.63
BIS	21.53 (4.30)	22.00 (3.84)	<1	0.67

### 3.3 SEM models

A multigroup structural model was estimated to analyze whether the hypothesized model (Fig. 1) might differ between pathologic and non-pathologic BW. The model had an acceptable fit (χ2 = 1181, df = 198, p = 0.001; CFI = 0.86, TLI = 0.81; RMSEA = 0.06, SRMR = 0.07).

**Fig. 2:**
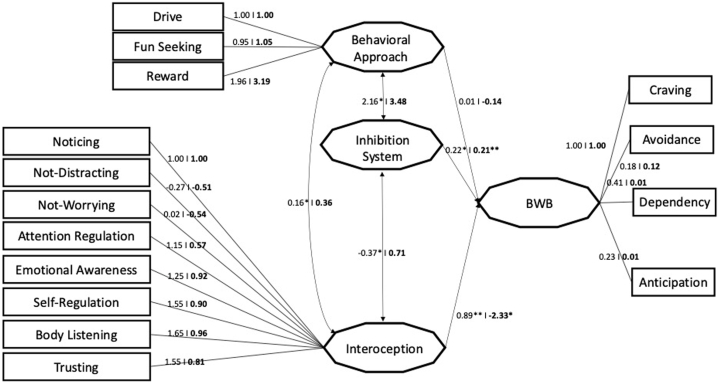
Path model in BW as leisure activity and problematic BW Legend for Fig. 2. The first coefficient for each variable refers to Leisure Activity, the second coefficient (in bold) refers to Problematic BW model. ∗ refers to significant indices.

Fig. 2 shows the significant paths in the model for participants who adopted BW as a leisure activity and BW as an at-risk behavior. In the first case, BIS system (estimate = 0.22; p = 0.001) and Interoception (estimate = 0.89; p = 0.01) were positively associated with BW, while BAS was not associated with BW (estimate = 0.01; p > 0.05). BIS, BAS, and Interoception were related (all p < 0.001); specifically, interoception was negatively associated with BIS (estimate = −0.37) and positively related to BAS (estimate = 0.16), while BIS/BAS systems were strongly related (estimate = 2.16). Considering the path model of BW as an at-risk behavior, the most notable difference between problematic and non-problematic BW was for the direct relationship of interoception to BW, which changed and became negatively related in Problematic BW (estimate = −2.33). In addition, for at risk behavior, the relationship between Interoception and Inhibition System was not significant.

## 4 Discussion

Interoception encapsulates and condenses internal bodily signals through neural and humoral pathways [3,6,9]. As a function useful for homeostatic balance (autonomic and neuroendocrine) and more complex allostatic responses, interoception affects adaptive behavior. The influence on behavior is exerted through the experience of motivational and emotional feelings, which coordinate physiological changes across body systems. Given these premises, this study tested the interoceptive-motivational hypothesis in the field of risk behavior to confirm the possible association between these systems in influencing the behavioral continuum from adaptive to maladaptive. We focused on analyzing the interdependence between body signals sensitivity and behavioral activation/inhibition systems, indices of homeostatic balance and motivational networks, respectively, which may characterize the expression of risk behavior [10].

The results of this study allow inferences about the still under-investigated relationship between interoception, motivational systems, and potential addictive behavior, and provide interesting insights into the BW phenomenon and its characteristics. Preliminary analyses confirmed an association between the interoceptive indices and BW. Specifically, the association emerged considering the emotional reaction and attentional responses to bodily sensations (i.e., the Not-Worrying and Not-Distracting scales), the ability to regulate attention (i.e., the Attention Regulation scale), the awareness of bodily sensations (i.e., the Noticing scale), the awareness of mind-body integration (i.e., the Self-Regulation and Emotional awareness scales), and the trust toward bodily sensations (i.e., the Trusting scale). Correlational results suggest that BW would be a strategy to reduce discomfort driven by body perception [25] or, in a bidirectional perspective, it may alter body signals and, consequently, adaptive interoceptive response in interaction with other cognitive dimensions may further enhance the approach. From a functional point of view, this association may be ascribed to a possible overlap of the neural networks of interoception and behavioral outcomes. A core center for this overlap may be the insula that emerged as the primary neural hub of interoceptive processing [6]. It is involved in the representation and awareness of physiological states, but also in emotional experience and decision-making [26]. On the other hand, the association between interoception and at-risk behavior is included in the framework of psychophysiological issues. As mentioned above, different studies have tested the role of hyperarousal in behavioral addiction, using indices such as blood pressure and heart rate [25]. In a recent study, Kennedy et al. [25] assessed the parasympathetic balance and emotion regulation in gambling disorder through respiratory sinus arrhythmia. The results of the study suggest a different parasympathetic pattern characterized by lower HRV in gambling and no differences in interoceptive accuracy as measured by the heart counting task. Autonomic response and bodily arousal consequent to the enactment of pleasurable behaviors can translate into conscious pleasure through shared networks with both emotional and interoceptive aspects.

However, this evidence suggests focusing on other variables that play a role in the relationship between bodily signals and behavioral outcomes. Accordingly, previous research suggests that anomalous BIS/BAS activity is associated with substance use [27], global levels of substance use expectancies and expectancies and substance use [28]. Moreover, studies have indicated an association between BIS/BAS and behavioral addiction, suggesting that similarities between substance and behavioral addictions involve the motivational domains to some degree [14]. The observed association, especially on the BIS, appears to contribute to the knowledge about risk and motivational systems that could be explained in terms of arousal and anxiety. High scores on the BIS scale cause individuals to feel anxious [29]. Feeling anxious and avoiding risky consequences with a high BIS is more likely to play a predictive role in binge-watching.

Correlational results are not confirmed by the analysis of variance, suggesting similar features in the interoception and motivational dimensions between the two groups, except for body cue perception. The absence of difference between the groups appears to confirm that it is an interaction between different aspects at determining the enactment of compulsive and potentially problematic behaviors in a continuous way rather than a difference in a single variable between two different expressions (categorial) of the behavior.

Despite the group with problematic BW recorded higher scores, no significant difference was found suggesting that the relationship between interoceptive sensitivity and BW is not direct and that there may be other aspects of interoception that more accurately detect these differences. These findings suggest a better perception and interpretation of body signals in individuals who have adopted BW as a pleasurable activity compared to individuals who exhibit a problematic and at-risk of addiction pattern of BW. However, this result is not unexpected, as the variables involved in this relationship mutually influence each other regardless of the pleasantness/compulsivity of the enacted behavior, suggesting a core feature of goal-directed behavior, in line with neurocognitive theory [26,30]. Accordingly, to verify the hypothesis about the possible role of the interaction between motivation and interoception as a marker of different behavioral outcomes, we tested a structured model by considering the two groups separately.

As hypothesized, our results suggest an intricate texture between interoceptive sensitivity and awareness (MAIA indices), motivational response to behavior (BIS/BAS), and BW behavior. Indeed, although an overall predictive role of both interoceptive awareness and behavioral avoidance of negative consequences (inhibition system) was highlighted, a different pattern emerges when BW is considered as a leisure activity or an at-risk behavior.

When BW is practiced as a leisure activity (i.e., increasing psychological well-being and negative affectivity; [15,19], a link between the motivational systems and interoception has been highlighted, indicating a complex direct and indirect burden of the physiological and psychological levels that can affect behavioral expression. In this sense, the adequate interpretation of the physiological activation (arousal) involved in the motivational approaches via interoceptive awareness would allow the regulation of the behavioral outcome, reducing the risk of occurrence of risky behavior and keeping it in an adaptive and functional range. One of the main human needs is the urgency to avoid harmful events and satisfy physiological exigences [31]. In the frame of behavior, people research allostasis (i.e., stability through change) through an efficient regulation of the body that anticipates and satisfies physiological needs before they arise [32]. To this end, the brain generates an internal predictive model of self and body in the world [33] that is compared to interoceptive processes. Discrepancies between top-down (i.e., brain model) and bottom-up (i.e., interoceptive model) representations generate prediction errors that affect individual satisfaction [34]. Consistent with the free energy principle [35], the attempt to minimize errors and increase consistency across models can follow multiple strategies that include both adaptive and maladaptive approaches [33]. In this framework, while watching TV series as a leisure activity, people enact the behavior to reduce distress and avoid negative feelings (inhibition system), and the interoceptive sensitivity to these feelings is useful to monitor and control the behavioral phenotype both directly and indirectly - via the association with the BIS/BAS systems. This would ensure the overlap of the internal and perceptual models, and the behavior would take on pleasant and positive connotations.

This consideration also helps to interpret the results regarding the range of problematic BW in which the pattern of association changes. Interoception and BIS/BAS lose their interaction in problematic BW, suggesting that at-risk behavior would result from non-communication between central and peripheral systems that may contribute to the discrepancy between internal and perceptual models. More specifically, the lack of connection between interoception and motivational dimensions would affect the processes behind the free energy principles [35], causing dysfunctional behaviors. The attempt to avoid the negative consequences is still a driver of the behavioral expression, even when it may take the features of a behavioral addiction. However, interoception would monitor BW, and if there are good levels of interoceptive awareness and sensitivity, the risk of occurrence of problematic BW is reduced. On the contrary, if the interoceptive system is altered, it may contribute to increasing the risk of behavioral addiction, in line with the evidence on the role of interoception in increasing self-control and reducing craving in addictions [10]. In this sense, compulsive behavior and the resulting addiction-like approach could be considered as a dysfunctional process of the whole system. The compulsive watcher becomes the embodiment of an unhealthy and unpredictable model, tailored to the problematic activity of feeding the negative habit, driven by impulsive aspects and disregarding bodily, physiological, and cognitive signals. The suggestive interpretation provided by these models fits well with the vision of the positive role of some recreational behaviors, which increase psychological well-being and appear to be more protective for the individuals’ health than simply not enacting the behavior at all [19,20]. However, in the continuum, complex and intricate mechanisms that cannot be observed and understood from only one perspective play a role in the risk of developing behavioral addiction.

However, some limitations should be noted. Concerning our sample, its convenient nature due to the dissemination plan, certainly does not make the results generalizable. Moreover, the low percentage of problematic BW in our sample (n = 16), although in line with the previous prevalence in the Italian population [16], reduces the statistical power and might affect the generalizability of your findings. Regarding our assessments, our measure focused on self-reported interoceptive sensitivity does not cover all interoceptive aspects, such as interoceptive accuracy. MAIA responses can vary according to individual awareness and personal interpretation, may be related to emotional state at the time of reporting, and may be subject to various biases (i.e., Social Desirability Bias, Recall Bias). Moreover, the scale may not adequately capture all nuances of bodily experiences. Accordingly, it is difficult to generalize findings to broader populations. Other domains of interoception may be more relevant to behavioral addiction, as reported in other studies [25], such as the interoceptive accuracy. Moreover, although our model captures a significant portion of variance in the data and provides a reasonable relationship among the variables, the marginal fit of our model suggests that some paths could be improved, additional variables could be considered, or other type of relationship should be explored (such as non-linear one). Accordingly, the inclusion of other psychological dimensions, particularly those related to emotion regulation, may help to better clarify the nature of the relationship between interoception and behavioral addiction. Also, personological characteristics (such as personality traits, coping strategies and life experiences) would affect the trajectories of the results. As well as adoption other methodologies, such as physiological measures (e.g., heart rate variability, skin conductance, pupillary responses) or behavioral assessments such as via collecting reaction and response times and accuracy in goal directed tasks focused on salient stimuli for the behavior, could improve our findings and validate our model in future studies also improving model fit. Finally, further studies should examine interoception and body-brain connections in other behavioral addictions or at-risk behaviors (e.g., internet, gaming).

## 5 Conclusions

In conclusion this study furnished interesting insight that should be considered from both researcher and clinicians to further explore behavioral addictions phenomenon and at-risk for addiction behaviors. considering the exploration of body-mind interaction, interoceptive ability to understand body signals and to interpret them, and motivational system that mediate the tendency to act or inhibit certain behaviors. According with this study, the characteristics of the integration between these variables, may furnish us a perspective to define at-risk and not-at-risk behaviors. The findings highlight that the interpretation and awareness of bodily signals and motivational systems, independently and in association with each other, could modulate behaviors, including the enactment of a compulsive approach. We can assume that the integration and interpretation of physiological signals by the interoceptive system are, to some extent, in interaction with the motivational features of the BIS/BAS model, managing affective responses and affecting the behavioral phenotype. The results of this study can be adopted to interpret the various behaviors that are enacted in modern society and may represent the expression of individual resources or risk factors. In this sense, BW can be considered a merely explanatory example conveniently adopted and useful to define the framework of functioning that could be generalized to other at-risk behaviors, providing more value to our results. Based on the findings of this study, the reasons why not-at-risk behaviors occasionally escalate into maladaptive behaviors, although not yet clearly understood, are surely affected by the complexity of the interactions between brain and body systems and how individuals attempt to cope with changes in these interactions. In this sense, preventive programs in the field of BA should surely consider not only the behavioral expression and its characteristics, but mainly the factors that determine and could cause the compulsivity, making it maladaptive. Increasing the awareness of bodily signals would prevent the management of stress generated by the negative context that would be associated with the motivation to approach the risk behavior. Moreover, our results can help clinicians in personalizing treatment plans. Interventions focused on interoception in the context of behavioral addictions aim to enhance individuals' awareness of their internal bodily sensations adopting different components, such as interoceptive exposure, somatic therapies, and mindfulness. Furnishing the tools to notice internal bodily sensations can help the individuals in adopting more appropriate behavioral responses, attuned in case of compulsive behaviors the early signs of craving, discomfort, or emotional dysregulation that may lead to the negative side of the behavior. By developing the ability to recognize these internal cues, individuals can interrupt the urge to engage in addictive behaviors. For instance, a clinician could help the individual identify distorted thoughts that lead to binge-watching (e.g., "I need to watch this entire series now to feel better"), and then guide them to notice how their body feels during these urges (e.g., rapid heart rate, tightness in the chest).

Certainly, these results should be considered preliminary, and many studies are needed in this regard, considering the interoceptive accuracy and not just the self-reported sensitivity, as well as including more complex models to define motivational frames. However, this study provides further support to the continuum hypothesis from pleasantness to the addictiveness of the behavior.

## CRediT authorship contribution statement

**Giuseppe Forte:** Writing – review & editing, Writing – original draft, Methodology, Formal analysis, Conceptualization. **Francesca Favieri:** Writing – review & editing, Writing – original draft, Methodology, Formal analysis, Conceptualization. **Francesca Agostini:** Writing – original draft, Investigation. **Maria Casagrande:** Writing – review & editing, Writing – original draft, Methodology. **Renata Tambelli:** Writing – review & editing, Supervision, Funding acquisition.

## Ethics

The project was approved by the Ethics Committee of the Department of Dynamic and Clinical Psychology and Health Studies (protocol code: 0000801). All methods were carried out in accordance with relevant guidelines and regulations and in line with the Declaration of Helsinki.

## Data availability statement

Data is available by contacting the corresponding authors.

## Founding sources

None.

## Declaration of competing interest

The authors declare the following financial interests/personal relationships which may be considered as potential competing interests:Renata Tambelli reports article publishing charges was provided by University of Rome La Sapienza. If there are other authors, they declare that they have no known competing financial interests or personal relationships that could have appeared to influence the work reported in this paper.
